# Quinolones: Action and Resistance Updated 

**DOI:** 10.2174/156802609789630947

**Published:** 2009-08

**Authors:** Karl Drlica, Hiroshi Hiasa, Robert Kerns, Muhammad Malik, Arkady Mustaev, Xilin Zhao

**Affiliations:** 1 Public Health Research Institute, New Jersey Medical School, UMDNJ, 225 Warren Street, Newark, NJ 07103; 2 Department of Pharmacology, University of Minnesota Medical School, Minneapolis, Minnesota 55455; 3 Division of Medicinal and Natural Products Chemistry, University of Iowa, Iowa City, IA 52242

## Abstract

The quinolones trap DNA gyrase and DNA topoisomerase IV on DNA as complexes in which the DNA is  broken but constrained by protein. Early studies suggested that drug binding occurs largely along helix-4 of the GyrA  (gyrase) and ParC (topoisomerase IV) proteins. However, recent X-ray crystallography shows drug intercalating between  the -1 and +1 nucleotides of cut DNA, with only one end of the drug extending to helix-4. These two models may reflect distinct structural steps in complex formation. A consequence of drug-enzyme-DNA complex formation is reversible  inhibition of DNA replication; cell death arises from subsequent events in which bacterial chromosomes are fragmented  through two poorly understood pathways. In one pathway, chromosome fragmentation stimulates excessive accumulation  of highly toxic reactive oxygen species that are responsible for cell death. Quinolone resistance arises stepwise through  selective amplification of mutants when drug concentrations are above the MIC and below the MPC, as observed with  static agar plate assays, dynamic in vitro systems, and experimental infection of rabbits. The gap between MIC and MPC  can be narrowed by compound design that should restrict the emergence of resistance. Resistance is likely to become  increasingly important, since three types of plasmid-borne resistance have been reported.

## INTRODUCTION

The quinolones are broad-spectrum antibacterial agents that are receiving increasing attention as resistance develops to other compounds. Unfortunately, the quinolones are also losing their utility due to bacterial resistance, which creates a sense of urgency to develop new, more effective derivatives. As a result, biochemical insights continue to emerge, and we can now begin to discuss crystal structures of drug-target-DNA   complexes.   Our   understanding   of   intracellular quinolone action is also deepening. For example, evidence is accumulating  that  lethal  action  is  due  to  chromosome fragmentation  and  the  resulting  surge  in  reactive  oxygen species (ROS). While finding new quinolone derivatives has continued along conventional lines that seek low MIC, that effort is expanding to include identification of compounds having  good  activity  with  mutants  resistant  to  existing compounds. We expect studies with fluoroquinolone resistance  to  eventually  lead  regulatory  agencies  to  add  anti-mutant  properties  to  the  evaluation  of  new  compounds. These and other developments make an update of quinolone action and resistance timely.

We use the term quinolone in a generic sense that refers loosely to a class of inhibitors that includes naphthyridones, quinolones, quinazolines, isothiazoloquinolones, and related agents. These compounds have as their targets two essential bacterial enzymes, DNA gyrase (topoisomerase II)  [1] and DNA  topoisomerase  IV  [2].  The  two  enzymes,  each  of which contains 4 subunits (2 GyrA or ParC and 2 GyrB or ParE), act by passing one region of duplex DNA through another [3-6];  during  that  process,  the  quinolones  form complexes with enzyme and DNA [1, 7]. The DNA moiety in the complex is broken, as revealed by detection of fragmented DNA following addition of protease, ionic detergent (sodium  dodecyl  sulfate,  SDS),  or  both  to  quinolone-containing  reaction  mixtures  or  lysates  from  quinolone-treated  bacterial cells [1, 7, 8]. The complexes are called “cleaved” or “cleavable” to indicate the presence of broken DNA that is covalently attached to the enzyme at the 5’ ends. Chromosomal  DNA  remains  supercoiled  when  obtained from cells treated with quinolones at bacteriostatic concentrations,  provided  that  the  complexes  are  kept  intact  by omission of protein denaturants from cell lysis procedures [8].  The  presence  of  supercoils  indicates  that  the  DNA breaks  in  the  complexes  are  constrained  in  a  way  that prevents  the  rotation  of  DNA  ends  that  would  otherwise relax supercoils. However, when cells are treated with lethal drug  concentrations,  the  supercoils  are  absent,  indicating release of the DNA ends from the complexes. That release is expected to fragment chromosomes.

The hallmark of quinolone action is formation of cleaved complexes.  *In  vitro*,  the  complexes  block  movement  of replication   forks   and   transcription   complexes,  thereby inhibiting  bacterial  growth [9-11].  Lethal  action  arises  at higher  quinolone  concentrations  in  parallel  with  chromosome  fragmentation.  Thus,  bacteriostatic  action  and  rapid lethal effects are distinct.  By  normalizing  lethal action  to MIC, it is possible to minimize the contribution of factors, such  as  drug  uptake  and  efflux,  that  would  otherwise confound comparison of quinolones during studies of drug mechanism.

Cleaved  complexes  are  also  important  for  quinolone resistance, because the common resistance mutations interfere with drug binding [12]. However, quinolone resistance also arises from mutations that alter drug uptake, efflux, and structure [13-17]. Many of these mutations do not by themselves provide clinical resistance, but they may facilitate the stepwise accumulation of additional mutations [13, 18, 19]. Stepwise   resistance   distinguishes   the   emergence   of quinolone resistance from the all-or-none phenomenon seen for  rifampicin  with  *Escherichia  coli*  and  *Staphylococcus aureus*  [20]. It also  underlies use of  the mutant selection window  hypothesis  as  a  framework  for  suppressing  the emergence  of  resistance (the  hypothesis  maintains  that resistant mutant subpopulations are selectively enriched and amplified when drug concentrations fall in a range above the MIC for the susceptible population and below the MIC of the least  susceptible mutant  subpopulation,  a value  called  the MPC).  The  selection  window  can  be  used  to  formulate dosing regimens, to choose compounds for therapy, and to design new agents.

Below  we  turn  first  to  biochemical studies of  cleaved complex   formation.   Knowledge   gained   from   crystal structures is moving us toward an atomic description of the complexes, with current data appearing to require a two-step model. An underlying assumption of structural studies is that the  type  II  topoisomerases  have  very  similar  structures; consequently, conclusions drawn with one enzyme are often applied to others. While this assumption is generally sound, the enzymes differ; in the second section we discuss the C-terminal domains of  the GyrA and ParC proteins, regions where major differences between gyrase and topoisomerase IV  appear.  We  then  shift  to  biological  consequences  of cleaved complex formation: inhibition of DNA replication, chromo-some  fragmentation,  and  accumulation  of  ROS. Recent studies of resistance include support for the mutant selection  window  hypothesis  and  the  discovery  of  new quinolone-like  compounds  that  exhibit  excellent  *in  vitro* activity  with  mutants resistant  to  existing  quinolones. We conclude with an update on the three types of plasmid-borne fluoroquinolone  resistance.  Readers  interested  in  earlier reviews are referred to [21-27].

## CRYSTAL   STRUCTURES   AND   MODELS   FOR CLEAVED COMPLEXES

For many years our understanding of quinolone action has been based on crystal structures of GyrA fragments [28] and eukaryotic topoisomerase II [29]. Such studies describe the  portion  of  GyrA  and  ParC  involved  with  the  DNA breaks. Most of the attention focused on helix-4 because it is the location of amino acid substitutions generally associated with  quinolone  resistance  and  presumably  drug  binding. Since quinolones were not part of these structures, the work revealed little about the positioning of the drugs.

When the structure of a co-crystal of yeast topoisomerase II and DNA was solved [30], several striking features were seen. First, the topoisomerase forces a  150° bend in DNA upon binding to the G (gate)-segment of DNA [30]. Second, the central four base pairs of the binding site adopt an A-form conformation, whereas DNA at the outermost edges of the G-segment binding site is B-form. Third, large conformational changes of the enzyme take place upon its binding to DNA [30], a conclusion that supports earlier biochemical work [31]. The conformational change creates a catalytic site having  a  DNA  binding  surface  that  extends  across  both protein  protamers.  This  conformation  positions  the  DNA backbone near a reactive tyrosine and a coordinated magnesium ion thought to be part of the DNA cleavage reaction.

**Fig. (1):**
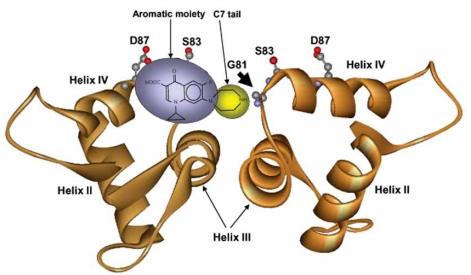
Helix-4 quinolone-binding model. The DNA-gate region is shown for a GyrA-GyrA dimer to which one fluoroquinolone molecule is bound such that the distal end of the C-7 ring (C7 tail) is near GyrA position 81 of one GyrA subunit; carboxyl and keto oxygens are near GyrA positions 87 and 83, respectively, of the other subunit.

Covariation  between  C-7-piperazinyl  ring  substituents and   susceptibility   to   particular   resistance  substitutions suggested a drug-binding orientation (Fig. **1**). For example, with *Mycobacterium smegmatis*, a fluoroquinolone with a C- 7-piperazinyl-N-linked ethyl moiety was less active against a Gly-81 to Cys variant (we use the *E. coli* numbering system for simplicity) than a similar quinolone with a C-linked ethyl [32]; amino acid substitutions at other positions in helix-4 failed to distinguish between the compounds. Since position 81 is located at the N-terminus of helix-4, the idea arose that the  C-7-distal  end  of  the  quinolone  binds  near  the  N- terminus of the helix. According to this hypothesis, the other (keto-carboxy) end of the quinolone would bind near amino acid  positions 83  and 87,  two  positions  where  major resistance substitutions map. As a further test of this idea, we recently   constructed   a   C-7   piperazinyl   N-bromoacetyl derivative  of  ciprofloxacin (Cip-Br)  that  has  intracellular properties consistent with crosslinking to Cys-81 (low MIC and irreversibility of inhibition of DNA synthesis that are specific  to  Cys-81  and  the  bromo  compound;  A.M.  and M.M.,  unpublished  observations).  Our  data  are  consistent with binding of quinolones to multiple points along helix-4 with the C-7 ring near position 81 (Fig. **1**).

**Fig. (2):**
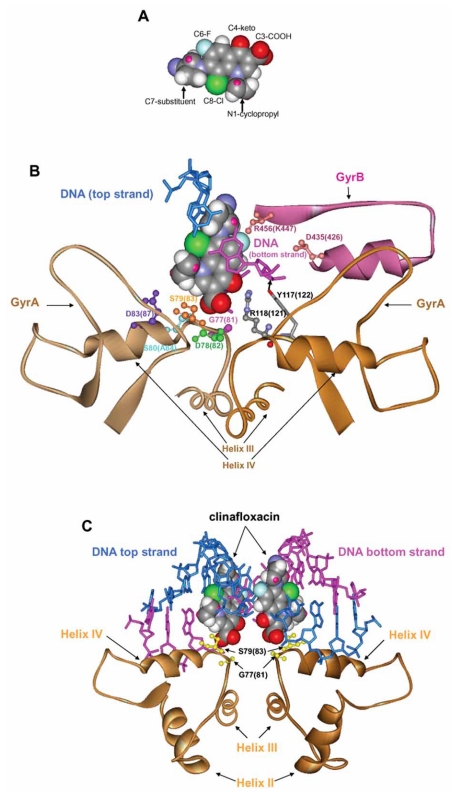
DNA intercalation model for quinolone binding derived from clinafloxacin-topoisomerase IV-DNA crystallography. Panel **A**. Space-filling model for clinafloxacin. Panel **B**. Relative arrangement of one clinafloxacin molecule, cleaved DNA, and portions of topoisomerase IV in the co-crystal structure described by [33]. Panel **C**. Relative arrangement of two clinafloxacin molecules, cleaved DNA, and portions of topoisomerase IV in co-crystal structure described by [33]. Protein and DNA residues in immediate contact with, or in close proximity to FQ are indicated (ball & stick representation). ParC features (helixes III, IV) are shown in beige; a short region of ParE (maroon) shows location of ParE resistance substitutions. DNA residues flanking the drug molecules are shown in a stick representation (top strand, blue; bottom strand, magenta).

A  very  different  idea  for  drug  binding  recently  arose from a crystal structure of a cleaved complex composed of the   DNA-binding   core   of   *Streptococcus   pneumoniae* topoisomerase IV complexed with broken DNA and either clinafloxacin (Fig. **2A**) or moxifloxacin [33]. In this model, each  fluoroquinolone  molecule  intercalates  in  the  gap between the -1 and +1 nucleotide pairs of the cleaved DNA bound  to  the  symmetrical  topoisomerase  IV  heterodimer (Fig. **2B**  shows  binding  of  one  clinafloxacin  molecule). Interaction  with  the -1  nucleotide  is  consistent  with  the observation  that  an  abasic  site  at  the  -1  position  inhibits formation of quinolone-induced cleaved complex at the site (HH, unpublished observations). A characteristic feature of the DNA intercalation model is the interaction of the C-7 substituent of the quinolone with DNA base pairs rather than with amino acid 81, which is far from the DNA moiety.

In the DNA intercalation model (Fig. **2B**), the 3-carboxyl group of the fluoroquinolone rests on a platform composed of the amino terminus of helix-4 such that the  3-carboxyl contacts Ser-79 (position 83 in *E. coli* GyrA) and is in close proximity to Ser-80 (Ala-84 of GyrA). The carboxyl group of Asp-78 (82 in GyrA) is not resolved in the structure, but it may  be  close  enough  to  the 3-carboxyl  group  of  the fluoroquinolone to allow formation of a Mg^2+^ bridge, which has been suggested to be important for drug binding due to the Mg^+2^-dependence of complex formation and reversal of DNA cleavage by EDTA [7]. Alternatively, the 3-carboxyl may  participate  in  an  electrostatic  interaction  with  the guanidine  group  of  Arg-118 (GyrA  121),  which  is  also unresolved  in  the  structure.  Finally,  one  of  the  hydrogen atoms of the guanidine group of Arg-118 can form a hydrogen bond with the  4-keto group of the drug, which would strengthen binding.

The DNA intercalation model shows how drug binding could  prevent  the religation  of  DNA. It also  explains the protective effect of some resistance mutations. For example, a  substitution  at  Asp-78 (GyrA 82)  would  eliminate  a putative  Mg^2+^  bridge,  thereby  weakening  drug  binding; substitution at Gly-77  (GyrA  81) could introduce a bulky side chain that would sterically clash with the oxygen of the fluoroquinolone 3-carboxyl group and/or push away the side chain of Arg-118, thereby interfering with interaction of this residue  with  the  fluoroquinolone.  The  close  proximity  of Arg-118 (GyrA 121)  to  the  fluoroquinolone 3-carboxyl group suggests that an interaction there might be significant to drug binding, which should be reflected in the recovery of resistance  substitutions.  Such  mutations  are  not  common, perhaps due to the importance of  the arginine residue for catalysis (this amino acid is highly conserved among type II topoisomerases). Substitutions of Ser-79 (GyrA 83), as well as those at position 80 (GyrA 84), are expected to reposition the drug molecule, thereby affecting other fluoroquinolone-protein  interactions. The DNA  intercalation  model  is also consistent with the protective effect of ParE substitutions at amino acids 435 (GyrB 426) and 456 (GyrB 447), which are located close to the bound drug.

The effects of several amino acid substitutions and drug structure  variations  are  unexplained  by  the  DNA  intercalation model. One example is the putative interaction of the C-7 substituent with position 81, as noted above. Another is  substitution  at  position 83  (GyrA  87),  which  is  highly protective  from  quinolone  action.  In  the  model,  GyrA-87 substitutions are  too  far  from  the fluoroquinolone-binding site to  interfere with drug action. The model also fails to explain effects of drug substituents at positions 1 and 8 that significantly  alter  both  the  antimicrobial  activity  and  the drug-target binding constant [34]: in the X-ray structure, the moieties at positions  1 and  8 make no contact with either DNA or protein. In particular, the  (-) isomer of ofloxacin (levofloxacin) binds to bacterial gyrase about  10-12 times more efficiently than the (+) isomer  [35], but modeling of ofloxacin onto the DNA intercalation structure provides no insight into the effects of the isomers. In addition, the role of the fluorine substitution  at position 6,  which  significantly improves antibacterial activity, remains obscure. Laponogov *et  al.* [33]  suggest  that  the  C-6  fluorine  might  influence charge distribution to favor stacking interactions with DNA bases. Finally, the protective effect of a substitution at Gly-77  (GyrA  81)  for  fluoroquinolones but  not nalidixic  acid [36] remains unexplained, as does the effect of the Cip-Br derivative when  Cys is substituted for Gly at position  81. The latter is particularly problematic, since the DNA intercalation model asserts that the C-7 piperazinyl ring, which we suggest can be crosslinked to GyrA Cys-81, stacks with DNA bases.

One way to accommodate the genetic-drug structure data described above with the intercalation model is to postulate that  quinolone  binding  is  a  multi-step  process  involving structures in which drug binding is quite different. Indeed, quinolone binding is known to involve at least two steps, one that occurs before DNA cleavage and one that occurs after [37,  38]. Thus, Figs.  **1**  and  **2**  may  describe  two  different steps in cleaved complex formation.

## DIFFERENCES    BETWEEN    THE    CARBOXYL-TERMINAL DOMAINS OF GYRA AND PARC

The  two  molecular  targets  of  the  quinolones,  DNA gyrase  and  DNA  topoisomerase IV,  are homologous,  and many interactions with quinolones are very similar for the two enzymes [25]. Indeed, all of the type II topoisomerases, except  for  topoisomerase  VI  of  *Sulfolobus  shibatae*,  are highly  conserved [21, 27].  However,  each  enzyme  also exhibits  a  distinct  catalytic  preference  that  reflects  its specialized intracellular function. For example, DNA gyrase is the only  enzyme that can  introduce negative supercoils into  DNA,  whereas  topoisomerase  IV  relaxes  negative supercoils  [39] and decatenates and unknots DNA  [40-42]. The binding  of  topoisomerase IV  to  the G-segment DNA takes  place  only  at  the  amino-terminal  catalytic  domain (NTD) of the ParC subunit, whereas GyrA binds to the G-segment at both its NTD and its carboxy-terminal domain (CTD).  Binding  at  the  CTD  is  thought  to  wrap  the  G-segment DNA around gyrase, thereby enabling the enzyme to catalyze DNA supercoiling [43, 44]. Thus, knowledge of CTD structure and function is important for understanding differences between gyrase and topoisomerase IV.

Recent structural studies show that a 35-kDa fragment of the CTD of *Borrelia burgdorferi* GyrA adopts a 6-bladed ‘β- pinwheel’ fold that is reminiscent of the  ‘β-propeller’ fold [45].  Three  other  CTDs,  the  *Bacillus  stearothermophilus* ParC CTD [46], the *Escherichia coli* GyrA CTD  [47], and the *E. coli* ParC CTD  [48], also adopt a β-pinwheel fold. While all GyrA CTDs possess 6 blades, ParC CTDs exhibit significant structural diversity  [45,  48]: some ParC CTDs, such as that from B. stearothermophilus, adopt a 6-bladed β-pinwheel fold, whereas others, such as the *E. coli* ParC CTD, adopt  a  5-bladed  fold.  Interestingly,  the  *B.  stearothermophilus* ParC CTD  superimposes well on the *E. coli* GyrA CTD  [48,  47]. These structural studies of GyrA and ParC CTDs,  together  with  phylogenetic  data  on  gyrases  and topoisomerase IVs, lead to a new picture of how bacterial type II topoisomerases are likely to have evolved  [45, 48]. Gyrase had been considered to be a specialized enzyme that had evolved primarily to supercoil DNA [21, 27]. However, the  specialized  function  of  topoisomerase IV,  incremental changes observed in the ParC proteins, and a wider distribution and greater conservation of GyrA than ParC, suggest that ParC CTDs are degenerate forms of the GyrA CTD and that topoisomerase IV evolved from gyrase [48]. The same conclusion  is  also  reached  by  comparing  GyrA  and  ParC NTDs [49].

Amino  acid  sequence  alignment,  supplemented  with secondary  structure  predictions,  reveals  that  the 7-amino-acid-long  GyrA  box,  QRRGGKG  [50],  is  the  only  motif unique to the GyrA CTD [45, 48]. Either deletion or alanine substitution in the GyrA box abolishes the ability of gyrase to   wrap   DNA   around   itself   and   to   catalyze   DNA supercoiling [51]. However, these mutations do not affect the relaxing  and  decatenating  activities  of  gyrase.  Thus,  the GyrA box is essential for the unique (supercoiling) activity of gyrase.

The  GyrA  CTD,  which  is  joined  to  the  GyrA  NTD through a flexible linker, moves between upper and lower positions during the catalytic cycle [52, 53]. The binding of the GyrA box to G-segment DNA may coordinate both the position of the GyrA CTD and the direction of G-segment DNA  bending  to  allow  gyrase  to  wrap  DNA  for  the supercoiling  reaction [51].  In  contrast,  the  *E.  coli*  ParC protein has an ordered linker between its NTD and CTD, and the position of the ParC CTD remains fixed relative to the ParC NTD. That prevents the ParC CTD from binding to G-segment  DNA  [48].  Instead,  the  ParC  CTD  captures  an incoming  T (transfer)-segment  DNA  to  catalyze  either decatenation or relaxation.

As discussed below, the wrapping of DNA turns out to be important to the stability of cleaved complexes formed with gyrase and the ability of the complexes to block replication fork  progression  [54,  55]. Thus, although  the GyrA  CTD does not directly interact with quinolone, the CTD appears to influence drug  action. Topoisomerase IV, which  does not wrap DNA, forms cleaved complexes that are intrinsically stable enough to cause replication fork arrest [54, 55].

## BACTERIOSTATIC ACTION OF QUINOLONES

### Replication  Fork  Arrest  by  Quinolone-Topoisomerase-DNA Complexes 

Rapid inhibition of DNA replication is one of the more striking  consequences  of  cleaved  complex  formation  with quinolones  [8,  56] and with inhibitors of eukaryotic DNA topoisomerases  [57,  58]. In  the case of  camptothecin  and eukaryotic  topoisomerase  I,  collision  of  replication  forks with cleaved complexes causes fork breakage and the release of  lethal  DNA  breaks [59].  Since  topoisomerases  share general features, the possibility arose that a similar phenomenon  would  occur  with  quinolones  and  gyrase/topoisomerase IV. Indeed, irreversible collision of replication forks with quinolone-mediated complexes was thought to be the primary source of cell death [60]. However, other work with quinolones   indicated   that   inhibition   of   replication   is reversible [56]. Thus, the relationship between inhibition of DNA   synthesis   and   cell   death   required   additional investigation.

Several   studies   have   been   performed   that   clarify relationships between cell death and collision of replication forks with  quinolone-mediated  cleaved  complexes. In  one approach, cells were treated in ways that block cell death, and  then  the  treatments  were  assessed  for  effects  on quinolone-mediated inhibition of DNA synthesis or growth. For example, chloramphenicol and anaerobic growth prevent first-generation quinolones from killing *E. coli.* The former has little effect on quinolone-mediated  inhibition of DNA synthesis [61],  and  the  latter  allows  quinolones  to  form complexes  that  block  growth,  presumably  by  inhibiting replication and transcription [62, 63]. Thus, replication and cell  death  are  distinct.  Another  approach  was  to  block replication by means other than quinolone treatment and then determine whether the quinolones still kill cells. When such an experiment was performed with a temperature-sensitive dnaB  mutant,  stopping  replication  had  little  effect  on  the lethal activity of the quinolones [64]. A third approach was to  reconstitute the collision  between  replication  forks and quinolone-containing  complexes  *in  vitro*  and  determine whether  double-strand  DNA  breaks  were  generated  or released  [9]. They  were not  [9].  Consequently, the  active DNA  breakage  and  reunion  activity  of  either  gyrase  or topoisomerase IV, which is required to arrest replication fork progression [9, 10, 54],  does  not  cause  the  breakage  of replication  forks.  Similar  conclusions  have  been  reached with topoisomerase II-targeting anticancer drugs using both *in  vivo* and  *in  vitro*  systems [65, 66].  We  conclude  that cleaved  complexes  composed  of  type  II  topoisomerases block replication fork movement without causing fork brea-kage and rapid cell death.

Since  quinolone-induced  cleaved  complexes  contain broken DNA, it was reasonable to assume that replication fork arrest would occur at any cleaved complex [22, 26, 67, 68, 69]. However, with *S. aureus* only a subset of quinolone- induced  cleaved  complexes  appears  to  be  physiologically competent for quinolone action [70], an observation that led us to examine how the stability of quinolone-induced cleaved  complexes  contributes  to  their  ability  to  block  replication fork movement.

One line of investigation showed that *S. aureus* gyrase requires high concentrations of potassium glutamate to wrap DNA, catalyze DNA supercoiling, and arrest replication fork progression *in  vitro*  [55]. Similar studies using an  *E. coli* mutant gyrase that lacks the entire GyrA CTD  (GyrA59), and  thus  cannot  wrap  DNA [43],  showed  that  gyrase-mediated  DNA  wrapping  is  required  for  replication  fork arrest [54]. Cleaved complexes formed with GyrA59 gyrase are also more sensitive to salt than those formed with the wild-type   gyrase,   and   GyrA59   gyrase-quinolone-DNA ternary  complexes  readily  disassociate  from  DNA.  Thus, wrapping of DNA is required for the formation of gyrase- containing cleaved complexes that are stable enough to block replication fork progression.

Topoisomerase  IV  also  forms  cleaved  complexes  that arrest  replication  fork  progression [54, 55],  but  topoisomerase IV does not wrap DNA. We found that quinolone- induced cleaved complexes formed with topoisomerase IV  are more stable than those formed with GyrA59 gyrase [54].  Critical differences may exist between the GyrA and ParC  NTDs with respect to their interactions with DNA and/or the quinolones in cleaved complexes. Another possibility is that  the placement of the CTD near the tower domain of GyrA or  ParC  is  important  for  the  stability  of  cleaved  complexes.  Gyrase uses DNA wrapping to position the GyrA CTD near  the tower domain [52, 53], whereas it is the ordered linker between the NTD and the CTD that places the ParC CTD  near the tower [48].

Studies  of  helix-4  also  bear  on  complex  stability.  As  pointed  out  above,  two  mutational hotspots for  quinolone  resistance, Ser-83 and Asp-87, locate on GyrA helix-4 [28],  and similar hotspots are found in analogous regions of ParC  [22, 68]. Thus, helix-4 is probably a part of the quinolone- binding site, as pointed out in the crystal structure section  above. Although DNA-binding domains, including helix-4, are highly conserved among gyrases and topoisomerase IVs,  differences do occur. Swapping helix-4 of *E. coli* GyrA with that of E. coli ParC [71] and swapping an extended region around helix-4 of *E. coli* GyrA with that of S. aureus GyrA [72] reveal that subtle differences in amino acid residues in and/or around helix-4 affect the quinolone sensitivity of a topoisomerase.   Furthermore,   quinolone-induced   cleaved complexes  formed  with  a  mutant  topoisomerase  IV  containing helix-4 of *E. coli* GyrA are more sensitive to quinolone and more stable than those formed with topoisomerase IV; they are less sensitive and less stable than complexes formed  with  gyrase  [71]. Thus, quinolone sensitivity  of a topoisomerase  correlates  with  the  stability  of  the  cleaved complex. Likewise, cytotoxicity of topoisomerase II-targeting anticancer drugs correlates with the stability of drug-induced cleaved complexes [73].

### Double-Strand DNA Breaks Arising from Inhibition of Replication

While we have argued above that lethal replication fork  breakage does not arise from collision of replication forks with cleaved complexes containing gyrase or topoisomerase IV, a small number  of  non-lethal  (repairable)  breaks probably arise. One line of evidence emerges from the signature response to quinolone treatment, the induction of the SOS regulon  [74-77]. Quinolone-mediated induction of the SOS response  requires  the  action  of  RecBCD,  which  in  turn requires a free DNA end to load onto DNA. Consequently, it is likely  that some double-strand  breaks are generated  by collision  of  replication  forks  with  cleaved  complexes. Indeed, in a plasmid model system where cleaved complex formation  blocked  replication,  double-strand  breaks  were observed [78, 79].

It has been suggested that the double-strand DNA breaks arising  after  replication  fork  stalling  are  generated  by  a recombination nuclease [78, 79]. Interestingly, a significant portion of the double-strand breaks associated *in vivo* with cleaved   complexes   and   blockage   of   replication   fork progression are reversible [79]. When replication forks are stalled  in  vivo,  they  trigger ‘replication  restart’  processes catalyzed by recombination proteins [80-83]. It is presently unclear  which  recombination  proteins  are  involved  in quinolone-induced generation of double-strand breaks. One candidate  is  the  RuvABC  complex.  Since  RuvAB  can reverse a topoisomerase IV-quinolone-DNA ternary complex *in  vitro* [84],  it  is  possible  that  RuvAB may  reverse  and dissociate the cleaved complex at a stalled replication fork before RuvC cleaves DNA to generate a double-strand break [79]. While we consider it important to distinguish the few DNA breaks associated with replication fork arrest from the extensive  chromosome  fragmentation  associated  with  cell death  (discussed below), it is conceivable that some repair proteins are involved in both.

## LETHAL ACTION OF QUINOLONES

### Quinolone Generations and Pathways to Cell Death

The quinolones kill *E. coli* by two pathways. One is blocked  by  inhibitors  of  protein  synthesis,  such  as  chloramphenicol, and by anaerobic conditions. The second pathway is  active  even  in  the  presence  of  chloramphenicol  or  the absence of oxygen. Four structural quinolone generations are distinguished   by   the   effects   of   chloramphenicol   and anaerobiosis  on  quinolone  lethality [63].  First-generation compounds,  such  as  nalidixic  and  oxolinic  acids,  are  not lethal  in   the  presence  of   chloramphenicol  or   during anaerobic growth; the second-generation agent norfloxacin fails to kill *E. coli* in the presence of chloramphenicol, but at high  concentrations  it  kills  cells  growing  anaerobically (norfloxacin  also  kills  *E.  coli*  suspended  in  saline,  while nalidixic  acid  does  not).  Ciprofloxacin,  a  third-generation compound, kills under both conditions but requires higher concentrations  during  anaerobiosis;  the  lethal  activity  of fourth   generation   C-8-methoxy   derivatives,   such   as PD161144, is affected little by chloramphenicol or anaerobic growth. These data fit with the idea that some compounds function more through one pathway than the other. The choice of pathway depends on quinolone concentration, since even the fourth-generation compounds are sensitive to chloramphenicol if quinolone concentrations are low enough.

The two lethal pathways are also observed in mycobacteria. For example, with *M. tuberculosis* gatifloxacin and moxifloxacin, two C-8-methoxy compounds, are indistinguishable when lethal activity is measured with growing cultures. However, when chloramphenicol is added, moxifloxacin is strikingly more active [85]. Moxifloxacin is also more active when growth of *M. bovis* BCG is arrested by treatment with nitric oxide [86]. Since the two fluoroquinolones differ only in their C-7 ring systems, we can begin to attribute death of non-growing cells to the C-7 substituent.

### Chromosome Fragmentation

Since    quinolone-enzyme-DNA    complexes    contain broken DNA and since chromosome fragmentation is likely to kill cells [87], we postulated that cell death arises from the release  of  DNA  breaks  from  protein-mediated  constraint existing in the cleaved complexes. The first evidence for this idea came from supercoiling studies with *E. coli* nucleoids, as pointed out in the introduction [61]. Treatment with lethal concentrations of oxolinic acid allowed relaxation of DNA supercoils,  an  event  that  failed  to  occur  when  cells  were pretreated with chloramphenicol. In these experiments high concentrations  of  ethidium  bromide,  a  DNA-intercalating agent, failed to introduce positive supercoils, indicating that the  DNA  relaxation  arose  from  DNA  breakage.  Ciprofloxacin,  a  compound  that  kills  cells  in  the  presence  of chloramphenicol, relaxed supercoils whether or not protein synthesis was blocked. This correlation between cell death and  chromosome  fragmentation  was  subsequently  strengthened by sedimentation and viscometric measurements [88].

Insight   into   the   chloramphenicol-insensitive   lethal pathway came initially from work by Ikeda. His laboratory found a form of quinolone-stimulated illegitimate recombination  that  was  attributed  to  gyrase  subunit  dissociation-reassociation  [89,  90]. Quinolone-mediated  gyrase subunit dissociation  could  explain  lethality  that  is  unaffected  by chloramphenicol: in the cleaved complexes the quinolones might pry gyrase subunits apart and fragment chromosomes. This  idea  is  supported  by  the  ability  of  gatifloxacin  to fragment isolated chromosomes in the presence of purified gyrase. Moreover, a GyrA A67S variant is killed by nalidixic acid in the presence of chloramphenicol [88], an event that does not occur with wild-type cells. In this variant an Ala residue expected to lie on the GyrA-GyrA dimer interface is substituted by Ser, a change that could weaken hydrophobic interactions  and  promote  subunit  dissociation.  So  far,  no structural  model  of  the  cleaved  complex  explains  lethal action.

The  basis  of  chromosome  fragmentation  that  requires ongoing protein synthesis is even less clearly defined. The three most obvious mechanisms for releasing DNA breaks from protein-mediated constraint are 1) protease digestion of gyrase, 2) nuclease-mediated cleavage on either side of the cleaved  complex,  and 3)  protein  denaturation.  Once  this fragmentation occurs, death arises from ROS, as described below.

### Amplification  of  Lethal  Action  by  Reactive  Oxygen Species

Collins and co-workers recently discovered that hydroxyl radical concentrations are elevated in *E. coli* following treatment  with  several  lethal  antimicrobials,  including  norfloxacin [91, 92]. We subsequently found that when both *sodA* and *sodB* were deficient, norfloxacin lethality was reduced. These   data   are   consistent   with   superoxide   dismutase normally promoting quinolone lethality, perhaps by stimulating   formation   of   peroxide [93].   A   deficiency   in catalase/peroxidase (*katG*) also elevated the lethal activity of norfloxacin, a result that was expected because a buildup of peroxide  should  lead  to  accumulation  of  highly  toxic hydroxyl radical [93].

Collins  *et al.* also  reported  that bacteriotstatic concentrations  of  thiourea  or 2,2’-bipyridyl,  agents  expected  to reduce  the  level  of  hydroxyl  radical,  inhibit  norfloxacin lethality [91].  That  led  to  the  conclusion  that  hydroxyl radical   contributed   to   quinolone   lethality [91].  Since inhibiting growth of *E. coli* is known to block norfloxacin lethality,   thiourea   and  2,2’-bipyridyl   treatment   was reinvestigated at subinhibitory concentrations. Even then the two agents interfered with norfloxacin-mediated killing [93]. Thus,  ROS  are  very  likely  to  play  a  role  in  quinolone-mediated lethality.

Since  norfloxacin  displays  a  complex  behavior  with respect to lethal action  [63], we reinvestigated the role of ROS   using   oxolinic   acid,   which   kills   only   by   the chloramphenicol-sensitive  pathway.  Like  chloramphenicol, thiourea  plus 2,2’-bipyridyl  almost  completely  blocks  the lethal action of oxolinic acid [94]. But only chloramphenicol blocks  chromosome  fragmentation (X.  Wang  and  X.Z., unpublished observation). Thus, the chromosome fragmentation   step   occurs   before   the   ROS   step.   Apparently chromosome fragmentation caused by oxolinic acid can be repaired, which explains the ability of inhibitors of ROS to almost completely block cell death  (in the next section we discuss a possible involvement of the Lon protease in repair). As  expected,  a  surge  of  hydroxyl  radical  accumulation follows oxolinic acid treatment, and that surge is blocked by chloramphenicol (X. Wang and X. Z., unpublished results).

Lethal  action  of  PD161144,  a  C-8-methoxy  fluoroquinolone  that  kills  *E.  coli*  by  the  subunit  dissociation pathway, is affected little by treatment with chloramphenicol or thiourea plus  2,2’-bipyridyl or all three agents together (X. Wang and X.Z., unpublished observations). These data further distinguish the two lethal pathways, and they suggest that  lethality  from  subunit  dissociation  is  independent  of ROS generation.

## LON  PROTEIN  AND  REPAIR  OF  QUINOLONE-MEDIATED LESIONS

Lon  protease  degrades  abnormal  proteins  and  proteins produced in  excess  [95]. By  targeting regulatory proteins, Lon  influences  a  variety  of  physiological  phenomena, including cell differentiation, sporulation, pathogenicity, and survival during starvation and anaerobic conditions. The Lon protein has ATPase activity, and part of the ATPase domain binds DNA. As a result, early studies identified Lon as a double-strand  DNA  binding  protein [96].  Large  DNA molecules  stimulate  both  the  ATPase  and  the  protease activities of Lon, which led to speculation that Lon might bind  chromosomal  DNA  adjacent  to  regulatory  proteins where it could control their turnover [97]. However, several *in vitro*  studies show  that the interaction of bacterial Lon with  large  DNA  molecules  lacks  nucleotide  sequence-specificity  [98]. Nevertheless, Lon remains a candidate for removal  of  proteins,  such  as  topoisomerases,  trapped  on bacterial chromosomes.

We first noticed a role for Lon protease in chromosome maintenance   when   examining   paradoxical   survival   of bacteria at very  high  concentrations of  quinolone  [99].  A deficiency of Lon protease eliminates paradoxical survival [100]. Plasmid-borne protease activity of Lon restores the paradoxical behavior of quinolones, while ATPase activity does not. These observations confirm that Lon is necessary for  paradoxical  survival  and  indicate  that  the  protease activity is indispensable.

To determine whether Lon affects chromosomal breaks in cleaved complexes, an empirical viscometric assay was applied to lysates of cells treated with various concentrations of nalidixic acid. When SDS was added to cell lysates to unfold chromosomes and release broken DNA from cleaved complexes, viscosity of lysates paralleled the lethal effect of nalidixic acid, initially dropping as nalidixic acid killed cells and then rising as high drug concentrations protected from death. In the Lon-deficient mutant, cell lysate viscosity was low  when  cells  were  treated  with  the  high  drug  concentrations that had been rendered lethal by the *lon* mutation. Thus,  cleaved  complexes  paralleled  bactericidal  effects, including those influenced by Lon.

Lon may also recognize and help repair other forms of cleaved complexes. One involves the derivative of ciprofloxacin  containing  an  N-bromoacetyl  C-7  piperazinyl group (Cip-Br). We expected the bromo substituent to form cross-links with a nearby cysteine, and bacterial strains having a Gly-81 Cys substitution in GyrA were exceptionally susceptible to Cip-Br. Moreover, inhibition of DNA synthesis by the quinolone was not reversed by washing cells with drug-free  medium  (M.M.  and  A.M.  unpublished  observations). However, lack of reversal by Cip-Br was seen only in a Lon-deficient strain, as if preferential recognition and removal of Cip-Br-Cys complexes by Lon obscured the irreversibility of putative drug-gyrase crosslinking.

A  third  example  of  Lon-mediated  protection  from quinolone was observed following treatment of *E. coli* cells with chloramphenicol, a bacteriostatic agent. With wild-type cells,  chloramphenicol  blocks  further  killing  by  oxolinic acid,  even  when  added  an  hour  after  the  quinolone. However, in Lon-deficient strains, chloramphenicol fails to rapidly  halt  quinolone-mediated  cell  death.  If  chloramphenicol is added before quinolone, the absence of Lon has no effect. These data are consistent with Lon being involved in the repair of lethal lesions formed by quinolones, lesions whose formation  is blocked  by  chloramphenicol. Whether Lon-mediated   repair   involves   direct   removal   of   the complexes or an indirect effect due to rapidly removing an unidentified lethal factor involved in fragmentation of DNA is not known.

Each  example  of  Lon  action  on  cleaved  complexes involves  a  situation  in  which  the  complex  may  have  an unusual structure  (extra drug molecules bound at the high quinolone  concentrations  that  allow  paradoxical  survival, cross-linked  drug-gyrase  complexes  formed  with  Cip-Br, and lethal, rather than reversible complexes after prolonged quinolone treatment). A Lon deficiency has no observable effect  on  nalidixic  acid-mediated  killing  in  an  otherwise wild-type  strain  at  low  to  moderate  concentrations  [100]. Under these conditions, Lon-mediated repair may be unable to compete with ROS-mediated killing.

We next turn to quinolone resistance. For many years, the prevalence of resistance was low for most pathogens, and the absence of plasmid-borne resistance was touted as one of the virtues   of   the   fluoroquinolones.   Heavy   medical   and agricultural use has negated both statements.

## MUTANT SELECTION WINDOW

### Stepwise Selection of Resistance Mutants

As with many antimicrobials, resistance to fluoroquinolones   is   conferred   by   genetic   variations   that   reduce intracellular  drug  concentration (e.g.  activation  of  efflux pumps) and/or reduction of the affinity of the compound for its  target. With  some pathogens, a single mutation, either chromosomal or  plasmid-borne,  is  insufficient for  clinical resistance.  In  such  cases,  it  is  often  the  accumulation  of multiple  changes  that  lowers  susceptibility  enough  to achieve resistance. If the initial drug concentration is low, non-target alleles will be selected, as seen with mycobacteria and *S. pneumoniae* [19, 101, 102]. If the initial concentration is moderately high, target mutations are selected  [19,  103, 104,  102].  After  the  population  acquires  one  mutation,  a second  emerges [105].  The  order  in  which  target  and nontarget alleles arise probably depends on the incremental increase  in  quinolone  concentration.  Repeated  cycles  of fluoroquinolone challenge, punctuated by periodic outgrowth of  pathogen  populations,  are  expected  to  cause  stepwise accumulation of mutations and therefore a wide variety of resistant mutants [14, 106].

The  gradual  accumulation  of  resistance  alleles  causes surveillance  studies  to  underestimate  the  emergence  of resistance, since strains can contain resistance mutations and still be considered clinically susceptible by MIC breakpoint criteria.   Those   mutations   increase   the   propensity   for attaining  additional  resistance  determinants  by  raising  the upper  limit  of  the  selection  window (discussed  below). Eventually strains accumulate enough mutations for MIC to exceed  the  resistance  breakpoint.  Dissemination  of  these resistant  mutants  can  then  cause  a  rapid  increase  in  the prevalence of resistance, as has been observed with *S. aureus* [107, 108].  Consequently,  resistance  can  appear  to  arise suddenly  even  though  the  early  stages  are  intrinsically gradual. To see the early stages it is necessary to perform a population analysis in which large numbers of cells from a culture are applied to antibiotic-containing agar plates and resistant colonies are counted [109]. The colonies that arise reflect the mutant subpopulations present in the culture.

### Mutant Selection Window Hypothesis

In the late 1990s we noticed that the recovery of myco-bacterial mutants from agar plates displays a characteristic response  to  fluoroquinolone  concentration [102, 110].  At low  concentrations,  the  drug  has  no  effect  on  colony formation  until  MIC  is  approached;  then  colony  recovery drops  sharply  as  susceptible  growth  is  blocked.  As  drug concentrations increase, a broad plateau is observed, since a variety  of  resistant  mutant  subpopulations  can  grow  and form colonies at those levels of drug exposure. Eventually a high  concentration  is  reached  at  which  colony  recovery drops sharply a second time. The second drop correlates with the MIC of  the least susceptible first  (single)-step  mutant subpopulation.  This  value  is  designated  as  the  mutant prevention  concentration  (MPC) because it severely limits the recovery of resistant mutants. At concentrations above MPC,  bacterial  growth  requires  the  acquisition  of  two  or more concurrent resistance mutations, which is a rare event. At low drug concentrations (slightly below MIC), selection pressure is greatly diminished because resistant mutants have no  growth  advantage  over  susceptible  cells.  Thus,  the selective amplification of resistant mutants occurs in a drug concentration range that is above MIC but below MPC. This drug  concentration  range  is  called  the  mutant  selection window.

### Experimental   Support   for   the   Selection   Window Hypothesis

Since  the  mutant  selection  window  was  derived  from static measurements, either with agar plates  [110] or with large volumes of liquid medium [111], it was important to determine  whether  the  window  also  exists  when  drug concentrations   fluctuate.   Measurements   with   *in   vitro* dynamic models show that the window can be observed with fluctuating  antimicrobial  concentrations  for  fluoroquinolones,  vancomycin,  and  daptomycin  [112-121].  It  is  also readily seen in rabbits infected with *S. aureus* and treated with  levofloxacin [122].  With  both  *in  vitro*  and  *in  vivo* experiments, static data fit well with dynamic measurements.

The  selection  window  hypothesis  differs  qualitatively from the  conventional  idea  in  which  the danger  zone for selection of resistant mutants lies below MIC  [123] rather than between the MIC and MPC [124]. The two ideas make different  predictions  about  the  emergence  of  resistance. According  to  the  conventional  view,  eradication  of  the susceptible population will suppress acquisition of resistance (“Dead bugs don’t mutate” [125]). In contrast, the selection window  hypothesis  maintains  that  resistance  can  emerge even when the susceptible population is eliminated, because resistant mutants may exist in a bacterial population prior to the  start  of  antibiotic  treatment.  Treatment  then  allows mutant enrichment  and  amplification. *In  vitro*  and  animal studies described above support the window hypothesis, as does a small clinical trial [126]. In the clinical study, newly hospitalized  tuberculosis  patients  were  screened  for  nasal colonization by *S. aureus* and then treated for tuberculosis using  a  protocol  in  which  rifampicin  was  the  only  agent active  with  *S.  aureus*.  After  several  weeks,  patients  were again sampled for *S. aureus* nasal colonization. In  92% of the  cases,  S.  aureus  colonization  was  eliminated,  which showed that the treatment was effective. The other 8% of the colonizing   isolates   became   rifampicin   resistant.   DNA analyses indicated that the resistant isolates evolved from the original, susceptible ones rather than from re-infection with different  strain  types.  Collectively,  these  are  the  results predicted by the window hypothesis for a situation in which drug  concentration  is  inside  the  selection  window  during treatment (MPC for rifampicin resistance is very high [20], which placed therapeutic concentrations inside the selection window).

### Lethal Action and Resistant Mutant Selection

One of  the predictions of  the  selection  window  hypothesis is that the emergence of resistance can be restricted by keeping  drug  concentrations  above  the  selection  window. This strategy  is based  on  blocking  mutant growth. Lethal action  is  an  added  effect  that directly  reduces susceptible pathogen numbers. That should help shorten treatment times, which in turn should reduce costs, toxic side effects, and the chance  that  new  resistance  will  develop.  Removal  of  the major population of susceptible cells should also increase the probability that host defense systems will eliminate resistant mutants.

Lethal  action  has  additional  importance  for  fluoroquinolones  having  gyrase  as  their  primary  target  because gyrase-mediated   resistance   is   genetically   recessive.   A recessive resistance mutation is not phenotypically expressed until the resistant, mutant protein has replaced most of the sensitive,  wild-type  copies (*E.  coli*  contains  more  than  a thousand gyrase molecules per cell and hundreds of cleaved complexes form on chromosomes  [8,  70,  127]). Until that time,  the  mutants  will  still  be  killed  by  the  quinolone. Consequently, compounds that are more lethal will be better at restricting the selection of newly formed resistant mutants. When  topoisomerase  IV  is  the  main  target,  resistance  is codominant 	[60];   consequently,   resistance   would   be expressed soon after the mutation occurred. In this situation lethal  action  would  not  have  as  great  an  effect  as  when resistance  is  recessive.  Recessive-dominant  considerations may partly explain why the frequency for obtaining target mutants  of  *S.  pneumoniae*  is 1,000  times  higher  for fluoroquinolones whose primary target is topoisomerase IV rather than gyrase [19, 128].

### Pharmacodynamics and the Selection Window

Some of the complexities of lethal action on the selection window  can  be  bypassed  by  empirical  PK/PD  considerations, since they take into account both bacteriostatic and bactericidal  activity. For  antimicrobial pharmacodynamics, the  efficacy  of  a  compound  is  commonly  related  to  two parameters,  its  potency  against  the  bulk  population  of  a particular pathogen, usually measured as MIC of the pathogen  culture, and  the concentration  achieved  at the site of infection. For the so-called concentration-dependent killers, such  as fluoroquinolones, the two  parameters are conventionally  combined  by  dividing  the  area  under  the  time- concentration curve in a 24-hr period (AUC_24_) by MIC. This pharmacodynamic  index (AUC_24_/MIC)  correlates  empirically  with  favorable  patient  and  microbiological  outcome [129, 130].  To  extend  this  idea  to  restricting  resistant subpopulation enrichment, MIC is replaced with MPC (the MIC of the least susceptible mutant subpopulation). Thus, a value of AUC_24_/MPC can be determined experimentally to define the upper boundary of the mutant selection window; that  value  takes  into  account  lethal  activity  of  fluoroquinolones  with  resistant  mutants [131, 132]  and  better defines  *in  vivo*  window  boundaries [122].  Consequently, treatment with lethal agents should require maintenance of fluoroquinolone concentrations above MPC long enough for killing to occur, but not throughout therapy, as would be the case  for  bacteriostatic  agents.  Experimentally,  restricted amplification  of  resistant  mutant  subpopulations  requires fluoroquinolone concentrations to be above MPC for only 20% of the dosing interval when *S. aureus* is treated with levofloxacin [122].

A general population-based approach has been developed to relate dose and patient outcome through measurements of AUC_24_/MIC [123, 133, 134]. The idea can also be used to evaluate  particular  doses  for  their  ability  to  restrict  the emergence of resistance [132]. Briefly, an animal model of infection is used to determine a target value of AUC_24_/MPC at which no resistance emerges. Then the ability of a given dose  to  attain  the  targeted  AUC_24_/MPC  with  a  human population  is  estimated  by  1)  determining  AUC_24_  for  the given  dose  using  a  patient  population, 2)  determining pathogen  MPC for  the  compound  using  isolates from the patient  population  to  be  treated,  and 3)  mathematically combining the population AUC_24_ and pathogen population MPC.  Due  to  the  pharmacokinetic  diversity  of  patient populations and susceptibility diversity of bacterial isolates, the output is the fraction of the patient population that will reach the pharmacodynamic target using a particular dose. Widespread use of this method requires additional measurements of  pathogen  population  MPC  [135-137]  since  MIC cannot be reliably used to predict MPC [138, 139].

### Practical Importance of the Window Hypothesis 

A major feature of the hypothesis is that it reveals one reason  why  emergence of  resistance  is  occurring:  clinical treatments place drug concentrations inside the window for long  periods  of  time.  That  facilitates  mutant  enrichment unless  host  defenses  eliminate  or  block  proliferation  of mutant subpopulations. It also provides a general approach for slowing the emergence of resistance: keep drug concentrations  above  the  window  or  use  combination  therapy. Applying  this  strategy  is  difficult  because  no  existing antimicrobial  has  been  developed  using  selection  window principles. Moreover, restricting resistance generally requires higher   doses   than   needed   to   cure   most   patients; consequently, toxic side effects become an issue. However, human pharmacokinetics for approved doses are known, and MPC has been  measured  for  many  drug-pathogen  combinations. Consequently, compounds can be compared for their ability to restrict the emergence of resistance.

## QUINOLONE-INDUCED QUINOLONE RESISTANCE

The   mutant   selection   window   addresses   selective amplification and enrichment of resistant mutants, but it does not consider the important property of mutant induction: the quinolones induce the mutagenic SOS response. To examine the effect of quinolone structure on the recovery of resistant mutants during drug exposure, we applied *E. coli* to  agar plates containing various compounds, and at daily intervals we counted the cumulative number of colonies  (this assay had  been  used  previously  to  assess  the  effect  of  various mutations on induction of  resistance  [140,  141]). With *E. coli*, colonies seen  after one day of  incubation  estimate a baseline  of  mutants  pre-existing  in  the  test  culture.  The number  then  increases  over  the  next  week.  The  rate  of mutant accumulation probably depends on a complex set of factors  that includes the rate at which  wild-type cells are killed and blockage of mutant growth  (M.M., unpublished observations).  A  methoxy  group  at  position  C-8  is  particularly restrictive, and a quinazoline-2,4-dione is much more effective than  its cognate fluoroquinolone. Thus, a simple agar-plate assay is available to compare compounds for the ability  to  restrict the mutagenic effects of  quinolones and related antimicrobials.

## NEW   QUINOLONE-LIKE   MOLECULES   HAVING ANTI-MUTANT ACTIVITY

Antimicrobial   development   conventionally   involves identifying new derivatives that are active against resistant mutants already enriched by earlier derivatives of the class. This  approach  keeps  the  clinician  one  step  ahead  of  the pathogen. Experience tells us, however, that many pathogens can easily make one mutational step. New compounds are likely to have a longer life span if they require pathogens to acquire  two  or  more  concurrent  resistance  mutations  for growth  in  the  presence  of  the  antibiotic.  If  the  mutant selection  window  is  closed,  *i.e.*  if  MIC =  MPC,  two mutational steps will be required for growth. Thus, a goal of quinolone  development  is  to  close  the  selection  window. That can be accomplished in two general ways. One requires a  compound  to  have  very  good  activity  against  resistant mutant  subpopulations;  the  other  involves  a  single  agent having two independent targets with similar susceptibility or two agents having independent targets. Examples using the two general approaches are described below.

### Closing  the  Selection  Window  with  a  Single  Agent Having a Single Target

MPC  is  the  MIC  of  the  least  susceptible,  first-step resistant  mutant  subpopulation.  MPC  may  be  difficult  to measure with some compounds and some bacteria because large numbers of organisms must be tested (on the order of 10^10^). As an initial screen, compounds can be tested for anti-mutant  activity.  This  activity  is  defined  as  the  MIC of  a known resistant mutant divided by the MIC of an isogenic wild-type  (susceptible)  strain. When  a battery  of  resistant mutants is examined, compounds can be compared for their ability to suppress the growth of mutants: compounds are sought that have MIC_mutant_/MIC_wt _ ≤ 1. Then it is necessary to measure MPC, since the battery of existing mutants may not have  accurately  represented  the  least  susceptible  resistant mutant  subpopulation.  For  example,  the  novel  compound being  tested  could  have  switched  targets  from  gyrase  to some other  enzyme. Such a compound might be effective against existing gyrase mutants, but it would not necessarily have  a  narrow  selection  window,  which  could  allow resistance to readily emerge.

**Fig. (3):**
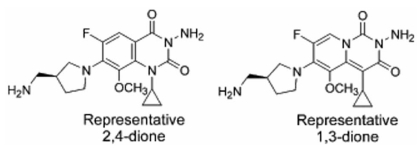
Representative 3-amino-8-methoxy-quinazoline-2,4-dione and 5-methoxy-pyrido [1,2-c]pyrimidine-1,3-dione structures.

Ellsworth  *et  al.*  showed  that  the  conversion  from  a quinolone core structure to a 3-amino-quinazoline-2,4-dione structure afforded  gyrase inhibitors  that  are active against known quinolone-resistant mutants of *E. coli, S. aureus*, and *S.  pneumoniae*  [142-146].  Structurally  similar  pyridopyrido[1,2-c]pyrimidine-1,3-diones   appear   to   possess   comparable activities [147],  and  we  recently  described  an  optimized synthesis of the 1,3-dione core ring system [148]. As a test of the anti-mutant approach, we prepared and evaluated a series of 3-amino-8-methoxy-quinazoline-2,4-diones (Fig. **3**) against quinolone-resistant *E. coli* mutants. By varying dione structure  at  the  N-3  and  C-7  positions,  we  were  able  to identify derivatives that brought the ratio of mutant to wild-type MIC close to unity [149], thus showing that optimized quinazoline-2,4-diones  drastically   reduce  the  protective effects of quinolone-resistant mutations in *gyrA* and *gyrB* of *E. coli*. Structural changes that lowered the ratio of mutant to wild-type MIC also lowered the absolute MIC. Moreover, the  most  bacteriostatic 2,4-dione  exhibited  rapid  lethality similar to the cognate fluoroquinolone when normalized to MIC   to   correct   for   drug   uptake/efflux [149].   When population analysis was performed with *E. coli* to examine the  ability  of  the  most  active 2,4-dione  to  restrict  the selection of resistant mutants, mutants were selected over a much narrower concentration range for the most active  8-methoxy-2,4-dione  tested  than  for  the  cognate  fluoroquinolone or for ciprofloxacin [149].

### Compounds Active Against More than One Target

**Fig. (4):**
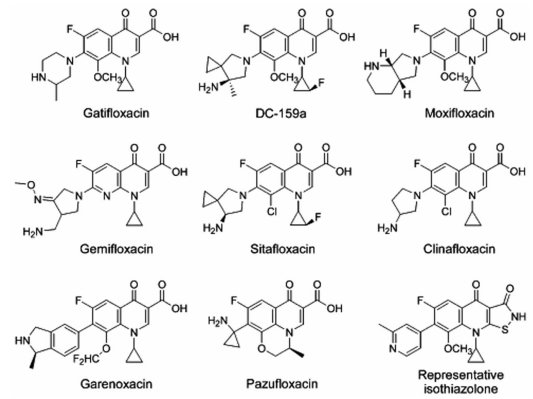
Representative newer quinolone-class antibacterial agents found to be equipotent or near equipotent inhibitors of both DNA gyrase and topoisomerase IV. It is notable that each structure differs from early generation fluoroquinolones (e.g. ciprofloxacin and norfloxacin) by having a position-8 group other than simple aryl hydrogen.

Some newer fluoroquinolones, des-fluoroquinolones, and other uniquely substituted derivatives target DNA gyrase and topoisomerase IV of certain organisms with near equipotent activity [150-154].  Analogs  of  other  quinolone-like  structural scaffolds, such as some heteroaryl isothiazolones (Fig. **4**),  also  show  equipotent  targeting  of  DNA  gyrase  and topoisomerase IV [155]. Consequently, a strain with a first-step  mutation  in  one  topoisomerase,  DNA  gyrase  or topoisomerase IV, would still be inhibited by action at the other target. When  the susceptibility of the two targets is equal, no selection window exists; for growth, a mutant must concurrently acquire mutations in both genes encoding the targets. The benefit of dual-targeting quinolones to slow the selection  of  quinolone-resistant  mutants  is  negated  when these agents are employed against *M. tuberculosis* and other organisms that lack topoisomerase IV.

Another approach to creating dual targeting compounds is to link members of different antibacterial classes. Indeed, conjugating two antibiotics to give multi-targeting agents has been studied by many groups with many antibacterial agents [156, 157]. With quinolones, this approach is exemplified by covalently   linked   rifamycin-fluoroquinolone   conjugates [158,  159]  and  oxazolidinone-quinolone  conjugates  [160]. Low  mutation  frequency  and  good  activity  against quinolone-resistant  gyrase  mutants  is  observed  because  the conjugates  are  derived  from  antibacterial  agents  having different molecular targets. The same approach can be used with separate agents in combination therapies. Variations in potency  of  the  individual  components  of  conjugates  and pharmacokinetic   differences   still   allow   emergence   of resistance, albeit at a slower rate.

## PLASMID-MEDIATED QUINOLONE RESISTANCE

Resistance  carried  by  plasmids  poses  two  threats  to quinolone efficacy. First, plasmids can transmit resistance to multiple  antimicrobials,  thereby  allowing  quinolone  resistance to be selected by use of other antibiotic classes and vice  versa.  Second,  plasmids  can  introduce  resistance determinants into  a bacterial population  at a much  higher frequency than occurs through spontaneous mutation  [161-163].  As  a  result,  resistance  is  expected  to  emerge  more rapidly  from  plasmid-borne  resistance  genes  than  from spontaneous  mutations.  The  practical  implication  is  that infections  caused  by  pathogens  containing  drug-resistant plasmids need to be treated with elevated drug concentration even  though  the  bulk  population  may  be  considered susceptible.

Three  forms  of  plasmid-mediated  quinolone  resistance have been identified. The first and best studied involves Qnr [164],  a  protein  that  interferes  with  quinolone  binding  to gyrase and topoisomerase IV. The second type expresses the quinolone-acetylating Aac (6')-Ib-cr enzyme that inactivates compounds  such  as  ciprofloxacin [165, 166].  The  third involves  an  efflux  pump  encoded  by  qepA  [167].  Of  the three, Qnr appears to have the most activity, increasing MIC up to 250 fold (QepA increases MIC by 10 fold [168] and Aac (6')-Ib-cr by 4 fold [165]). Below we focus on Qnr.

Qnr was discovered in a strain of *Klebsiella pneumoniae* exhibiting resistance to fluoroquinolones and 13 other agents [164]. The discovery  of  related  proteins  (QnrB  [169]  and QnrS [170]) subsequently caused the original protein to be renamed  QnrA.  QnrA  is  a 218-amino-acid  protein  that belongs to a large protein family characterized by pentapeptide  repeats (the  pentapeptide  protein  family  includes roughly 500  members  that  display  a  wide  variety  of properties [171, 172]). Insight into how the gyrase-protecting subclass might act came from structural analysis of MfpA [173], a Qnr homologue found in mycobacteria [174]. When MfpA was expressed in *E. coli*, purified, and crystallized, its three-dimensional structure revealed that the protein dimer has size, shape, and electrostatic similarity to B-form DNA [173]. The protein appears to be a DNA mimic. 

The  Qnr  proteins  lower  quinolone  binding  to  DNA complexes formed with gyrase or topoisomerase IV  [175]. Binding of Qnr to the two enzymes appears to be specific rather than a general protein-binding property, and it does not  require  quinolone,  DNA,  or  ATP [176].  Qnr  also reverses  quinolone-mediated  inhibition  of  the supercoiling activity  of  gyrase [176].  Even  a 1,000-fold  excess  of ciprofloxacin fails to overcome the Qnr-gyrase interaction; consequently,  Qnr  is  likely  to  act  by  altering  the  DNA-binding  properties  of  gyrase  rather  than  by  competitive binding to a quinolone interaction site  [176]. Since quinolone  resistance  arises  in  a  stepwise  fashion,  reduced susceptibility due to the presence of *qnr* is expected to be an important factor in  the emergence of resistance, either by adding to the effect of an existing resistance allele to render a strain clinically resistant or by serving as an early step in the pathway to resistance. As expected, increased MPC has been reported with Qnr-containing bacteria [13].

Often  plasmids  having  a  QnrA  determinant  also  carry genes that confer resistance to other anti-bacterials, such as aminoglycosides, 	β-lactams, 	chloramphenicol, 	and sulfonamides [177].  The  presence  of  multiple  antibiotic resistance genes on the same plasmid explains the frequent multidrug-resistant  phenotype  of  Qnr-positive  enterobacterial isolates. The fluoroquinolone-resistance plasmids are conjugative and carry both integrons and transposons [177]. They also have a broad host range: the plasmids have been obtained   from   a   variety   of   enterobacteria   including *Citrobacter freundii, C. koseri, Enterobacter aerogenes, E. amnigenus,  E.  cloacae,  E.  sakazakii,  E.  coli,  Klebsiella oxytoca,  K.  pneumoniae,  Providencia  stuartii,  Salmonella enterica, Serratia marcescens, Shigella dysen-teriae,* and *S. flexneri*  [178-181]. Moreover, Qnr-expressing plasmids are widely  distributed  with  respect  to  geography [177].  For example, they have been isolated in Bangladesh [182], China [183-185], France [186], Germany [187], Korea [180], Japan [170], Taiwan [188], Thailand [189], Turkey [190], United Kingdom [191], and the United States [181, 192]. Thus, we expect qnr to cause serious resistance problems.

The genes responsible for the other two types of plasmid-borne resistance have not been studied as extensively as *qnr.* The quinolone-acetylating Aac (6')-Ib-cr enzyme inactivates compounds  such  as  ciprofloxacin  by  placing  an  acetyl substituent  on   the  unsubstituted   nitrogen   of   the  C-7 piperazinyl ring [165]. The enzyme also lowers susceptibility to norfloxacin, which has the same C-7 ring as ciprofloxacin. However, it has no effect on quinolones, such as enrofloxacin, pefloxacin, levofloxacin, and gemifloxacin, that lack an unsubstituted piperazinyl nitrogen [193]. So far, bacterial isolates carrying the *aac (6')-lb-cr* gene have been recovered from China [165, 185], France [194], the United States [15], and Uruguay [195] in a variety of Enterobacteriaceae such as *C. freundii, E. cloacae, E. coli, and K. pneumoniae*  [195, 185]. The other plasmid-borne resistance factor, the QepA efflux pump, was first found in 2006 in a clinical isolate of *E. coli* from Japan [167]. MIC for hydrophilic fluoroquinolones, such as norfloxacin and ciprofloxacin, increases by 10 fold compared with plasmid-free counterparts [168]. So far, the prevalence of  QepA-mediated  resistance in  humans  is low (0.3%  among  *E.  coli*  isolates  collected  from 140 Japanese hospitals between  2002 and  2006  [167]; 0.8% of ESBL-producing enterobacterial isolates collected in France during 2007 [196]).

## CONCLUDING REMARKS

The  quinolones  continue  to  be  an  important  class  of antimicrobial agent. The reaction mechanism of the target enzymes is understood in considerable detail, and it is clear that  formation  of  drug-enzyme-DNA  complexes  is  the central event in quinolone action. However, our knowledge of  these  complexes  is far  from  complete. For  example,  a crystal structure of the complex has been reported [33], but an additional structure may be required to explain what is likely to be multistep binding [197]. Release of DNA breaks from  cleaved  complexes  and  the  resulting  chromosome fragmentation  continues  to  explain  lethal  action,  although with  the older  quinolone derivatives it is  clear  that death ultimately arises from the accumulation of hydroxyl radicals. How  the DNA  breaks are released  from protein-mediated constraint  and  how  they  promote  a  cascade  of  reactive oxygen  species  is  unknown.  The  newer  fluoroquinolones also  cause an  ROS  surge, but chromosome fragmentation appears to kill faster than ROS.

Bacterial  resistance  to  the  quinolones  is  a  growing problem [198-200]. Many aspects are now predictable within the framework of the mutant selection window hypothesis: continued   use   of   dosing   regimens   that   place   drug concentrations inside the selection window for long periods of time will surely erode the usefulness of the compounds. Whether  keeping  concentrations  above  the  window  sufficiently restricts the emergence of resistance remains to be seen, especially since plasmid-borne resistance is becoming widespread.  Assays  are  now  available  to  screen  new compounds for the ability to restrict mutant amplification, and new derivatives are emerging. Thus, the future remains bright for the quinolone class if the research developments are exploited judiciously.
